# Nutritional and microbiological effects of vermicompost tea in hydroponic cultivation of maple peas (*Pisum sativum* var. *arvense* L.)

**DOI:** 10.1002/fsn3.3299

**Published:** 2023-04-06

**Authors:** Xinyi Jiang, Ci Lu, Runmeng Hu, Wenyang Shi, Libang Zhou, Puzhao Wen, Yizhou Jiang, Yangming Martin Lo

**Affiliations:** ^1^ Institute for Advanced Study Shenzhen University Shenzhen China

**Keywords:** beneficial bacteria, hydroponics, plant growth, vermicompost tea

## Abstract

Hydroponics receives considerable attentions due to population expansion, soil pollution, and farmland scarcity. However, one significant problem is that its residual effluents are detrimental to the surrounding ecosystem. There is a dire need to find an organic, alternative, biodegradable substrate. Vermicompost tea (VCT) was investigated on its suitability as a hydroponic substrate to provide both nutritional and microbiological benefits. It was found VCT increased the biomass of maple peas (*Pisum sativum* var. *arvense* L.), increased stem length, raised the potassium ion content, and promoted the uptake of nitrogen by the roots. Meanwhile, the microorganisms associated with earthworm guts were detected in the maple peas root system, namely the inter‐rhizosphere of maple peas, including *Enterobacteriaceae*, *Pseudomonadaceae*, and *Flavobacteriaceae*. The presence of these microorganisms in large number indicated the ability for VCT to retain earthworm intestinal microbes via intestinal tract movement, excreting, and other vital activities. In addition, *Rhizobia* spp., such as *Burkholderiaceae* and *Rhizobiaceae* were also identified in VCT. They are critical for legumes as they can form root or stem nodule symbioses to produce growth hormone, vitamins, nitrogen fixation, and protection against plant stress. These findings are consistent with our chemical analysis that VCT‐treated maple peas had increased nitrate and ammonium nitrogen content relative to the control in roots, stems, and leaves, hence increasing maple peas' biomass. The abundance and species of the inter‐root bacterial population were found to change during the experimental period, indicating the importance of microbial balance to the growth and nutrient uptake of maple peas.

## Graphical abstract

Vermicompost tea (VCT) was investigated on its suitability as a hydroponic substrate to provide both nutritional and microbiological benefits. VCT increased the biomass of maple peas (Pisum sativum var. arvense L.), increased stem length, raised the potassium ion content, and promoted the uptake of nitrogen by the roots. The microorganisms associated with earthworm guts were identified in the maple peas root system, consequently promoting the growth of maple peas.

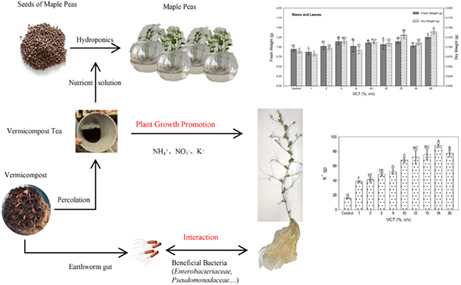

## 1 INTRODUCTION

Hydroponic cultivation has been the focal point for urban soilless agriculture in the wake of land scarcity and a growing population (Łaźny et al., 2022), and continues to gain popularity due not only to avoidance of contaminated soils while taking active control of plant nutrition and health (Vinci & Rapa, 2019; Walters & Stoelzle Midden, 2018), but also to save water and to reduce weight load on building structures (Arcas‐Pilz et al., 2022). However, the adverse impacts resulting from hydroponic production on surrounding natural ecosystems cannot be overlooked, namely the production, extraction, application, and disposal of fertilizers employed to secure direct plant nutrient uptake (Rufí‐Salís et al., 2020; Sanjuan‐Delmás et al., 2018). For instance, salt accumulation can occur in irrigation water even with a completely closed hydroponic system (Savvas et al., 2005); mineral wool, a solid substrate most widely used in hydroponic cultivation of tomato, bell pepper, and cucumber, emits substantial amount of CO_2_ into the environment while resulting in considerable burdens during disposal (Łaźny et al., 2022). There remains a dire need to search for alternative organic, biodegradable substrates that will provide the needed nutrients in support of improving plant growth without the aforementioned adversity.

The ability to recycle a wide variety of organic waste into an effective fertilizer abundant in macro‐ and micronutrients without causing soil pollution or degradation has made vermicompost (VC) an attractive option for sustainable agriculture (Arosha & Sarvananda, 2022; Ghaffari et al., 2022; Makode, 2015; Srivastava et al., 2018). The presence of VC in soil, through active interactions between earthworm and microorganisms, has long been shown to not only act as a soil conditioner that provides nutrients to plants, reducing the carbon to nitrogen ratio, but also improve soil texture by increasing soil porosity and water holding capacity, thereby reducing tillage and irrigation (Datta et al., 2016; Sharma & Garg, 2018; Van Groenigen et al., 2019). It is well documented that VC promotes the growth of microbial communities in the soil, including nitrifying bacteria (Powers, 2018), phosphorus‐enhancing bacteria (Yatoo et al., 2020), humic acid, and defense enzymes that promote plant growth, development, and productivity (Adiloğlu et al., 2018; Olle, 2016). Tognetti et al. (2005) conducted a comparative study of municipal waste compost and VC, and they observed that VC gave higher ryegrass yield when applied at the same rate of 20 and 40 g/kg, which could be due to the presence of higher nutrient concentration and microbial activity. Several authors have also reported increased growth and yield of plants such as mung beans and sage with VC compared to compost (El‐Haddad et al., 2020; Soobhany et al., 2017). VC is considered to have potential as a bio‐organic fertilizer for the faster early growth of flowerbed plants when used as a component of soilless greenhouse container media (Atiyeh et al., 2000). The processes through which earthworm casts become more fertile than soil were conceptualized by Van Groenigen et al. (2019) into two pathways, namely (1) through *concentration* of existing fertile components by preferential feeding, or (2) through *transformation* that is associated with gut passages, resulting from a wide variety of microorganisms that are involved in numerous biochemical processes (Drake & Horn, 2007).

Vermicompost tea (VCT), the aqueous extract of VC (Edwards et al., 2011), presents itself as an opportunity to modern urban agriculture, that is, hydroponic cultivation. It requires a relatively small number of earthworms to cover a large production area while extracting all the desirable biochemical properties of solid VC. It has been shown that VCT also contains microorganisms, nutrients, and plant growth promoters that are beneficial to plants by foliar application or by adding it to the soil (Abdel Salam & Roshdy, 2022). Applications of VCT have been shown to overcome nutritional deficiencies in high‐yielding intensive crop production where organic materials are the sole nutrient source (Ruiz & Salas Sanjuan, 2022), as evidenced by enhanced seed germination, plant growth, increased yield, and suppression of plant diseases by inducing plant resistance to pathogens, or producing direct toxicity to plant pathogens (Kim et al., 2015; Koné et al., 2010; Martin & Brathwaite, 2012). Researchers in Australia found that plants grown on VCT exhibited growth parameters exceeding those of plants grown on commercial hydroponic solutions, suggesting that VCT is an ecologically friendly alternative to growth substrates in terms of quality (Ansari et al., 2015). Equally noteworthy is that the coelomic fluid (CF) of the earthworm *Eisenia fetida* has been found to contain bioactive compounds with antifungal and insecticidal properties (Gudeta et al., 2022), which, inherently, could be an effective factor in resisting pests and suppressing diseases when VCT is used hydroponically. The use of VCT as a pest‐resistant agent in hydroponically grown plants reduces the residue of chemical compost in the environment.

In addition to the nutritional, antifungal, and pest‐resisting aspects, the presence of inter‐root microorganisms is an essential component of plant growth. It has been noted for decades that the soil surrounding plant roots is very rich in microorganisms, and thus is termed “inter‐root” (Bashan & Holguin, 1995; Hartmann et al., 2008). These microbes have been studied extensively for their role in plant health, and as more is learned about the processes that occur in the inter‐roots, these relationships can begin to be leveraged to increase plant growth in an environmentally sustainable manner (Lin et al., 2017; Ma et al., 2022). Harnessing the ability of microbes to provide essential micro‐ and macronutrients to plants is an ongoing goal of inter‐root plant microbial research (Fei et al., 2022; Tkacz & Poole, 2015). Inasmuch as plant roots are critical for nutrient acquisition and productivity, however, most literature knowledge remains limited on the microorganisms in the soil, leaving a void about the inter‐root microbial communities in hydroponic cultivation. Therefore, the present study aimed to investigate the effect of VCT as a hydroponic substrate on the growth of maple peas. Also elucidated were the corresponding changes in beneficial bacteria in the plant root system in an aqueous environment.

## 2 MATERIALS AND METHODS

### 2.1 Vermicompost tea (VCT) and hydroponic experiments

The experiment was conducted in the ecological reintegration laboratory of Shenzhen University, Shenzhen, China. The vermicompost was obtained from “Taiping II” (*Eisenia fetida*) earthworm raised in a wooden earthworm tower established by our lab for 30 days and fed with untreated vegetables from campus canteens. The tap water used for VCT preparation was exposed to sunlight for 4–5 days to remove residual chlorine. Ca. 24 g of earthworm casting was added to each liter of treated tap water prior to addition of 2 tablespoons of brown sugar and mixed well. The finished VCT was left to stand for 24 h.

Maple peas (*Pisum sativum* var. *arvense* L.) were used as a model plant to test the effect of VCT. They were purchased from a commercial vendor (Shui Sheng Tian, Weifang, Shandong, China). All maple peas were soaked in pure water for 24 h prior to use. Seeds of similar size (10 seeds each batch) were selected for germination in respective hydroponic solutions (10 mL) containing VCT at 0% (control in following), 1%, 2%, 5%, 8%, 10%, 12%, 15%, 18%, and 20% of the total volume. The seed‐containing VCT solutions were covered by moist newspaper to create darkness for 48 h. All growth experiments were conducted under room temperature (25°C) and 85% humidity and repeated three times. The total of 10 germinated maple peas (all with similar root length) were selected from each concentration for hydroponic experiments up to 26 d.

### 2.2 Chemical analysis

The pH and electrical conductivity (EC) were measured with FiveEasy Plus pH meter FE28 and FiveEasy Plus EC meter FE38 (Mettler Toledo). Dissolved oxygen (DO) was measured by an electrochemical probe (HJ 506–2009). The total nitrogen and phosphorus of the hydroponic substrates were detected using a TU‐1810 UV spectrophotometer (Beijing General Analytical Instrument). The hydroponic substrates were filtered through 0.22 μm membranes and then assayed for potassium, calcium, sodium, and magnesium ions using a Thermo Fisher iCAP 7400 (Thermo Fisher Scientific).

### 2.3 Plant physical and chemical properties

The ammonium, nitrate nitrogen, and total carotene in the stems, leaves, and roots of germinated maple pea leaves were determined using a TU‐1910 UV spectrophotometer (Beijing General Analytical Instrument) (Lv et al., 2004; Zhang et al., 2000). The potassium and selenium contents were determined by a Thermo Fisher iCAP 7400 dual‐channel inductively coupled plasma emission spectrometer (ICP‐AES) and a Thermo Fisher iCAP RQ inductively coupled plasma mass spectrometer (ICP‐MS) (GB/T 35871–2018). An LA‐ST10 root image analyzer (Wuhan Greenpheno Science and Technology) was employed to measure the total root length of maple peas with sample roots laid flat on the scanning platform while appropriate amount of water was added for scanning. The stem length of the maple peas was measured and analyzed via ImageJ software (National Institutes of Health).

### 2.4 DNA extraction and 16S rDNA assay

Residual microbial DNA in each of the hydroponic solutions after growth experiments was extracted with a DNeasy PowerWater Kit (100) (Qiagen) according to the manufacturer's instructions. Maple pea roots' DNA was extracted on days 7 and 26 with a MagAttract HMV DNAkit (Qiagen) according to the manufacturer's instructions. High‐throughput 16S rDNA gene sequencing was performed utilizing the Illumina HiSeq platform by Novogene Co. (Beijing, China). The V3‐V4 region was amplified with the 338F/806R primer set, and QIIME (v1. 80) (Bolyen et al., 2019), ImageGP (Chen et al., 2022), and R language were used for result mapping.

### 2.5 Statistical analysis

All experimental data were processed with IBM SPSS Statistics 2.2 software (IBM). The one‐way analysis of variance (ANOVA) method was selected to obtain the mean and standard deviation of each data group. Multiple comparisons were performed using the Waller–Duncan method, and the growth indicators of maple peas were analyzed at a 99% confidence level (*p* < .01). The alpha diversity of microorganisms was analyzed at a 95% confidence level (*p* < .05).

## 3 RESULTS AND DISCUSSION

### 3.1 Chemical properties of VCT at different concentrations

Testing the chemical indicators of the of VCT at different concentrations (Table 1), the initial solution had a similar pH, close to neutral, while the VCT itself was more alkaline. Conductivity increased with increasing VCT concentration, but all were within the range required for optimal maple peas growth. Nitrogen, phosphorus, potassium, and other micronutrients were higher in the VCT at different concentrations than control (0% VCT). These trends were all proportional to the concentration of VCT. After 26 days of germination, hydroponic media from plants germinated with varying VCT concentrations were collected and the total nitrogen and potassium ions were measured because nitrogen and potassium ions have a significant influence on the growth of plants. Nitrogen (N), as a macro element, plays a vital role in the growth and development of plants. Deficiency in N would reduce plant productivity by reducing photosynthesis, leaf area, and leaf lifespan. Following 26 days of incubation (Table 2), the solutions with 1% and 2% VCT content had an acidic pH (pH <6), while the rest of the experimental groups remained in the neutral range. The EC of all groups remained in the range suitable for maple pea growth. The 20% VCT had a higher EC, which may affect maple peas growth. Total nitrogen content accumulated in the experimental groups with high concentrations (15%, 18%, 20% VCT), and higher nitrogen content would promote roots growth while causing slow stems and leaves growth (Tian et al., 2021). The low concentration (1%, 2%, 5%) groups had low total nitrogen. The potassium ion contents were all lower than the initial concentrations, indicating that maple peas have a demand for potassium ions and absorb more.

**TABLE 1:** Various chemical properties of VCT at different concentrations on day 0.

VCT (%, v/v)	pH	EC (ds m^−1^)	DO (mg L^−1^)	TN	TP	K^+^	Ca^2+^	Na^+^	Mg^2+^
Control	6.84	0.22	0.24	2.87	0.13	5.29	17.90	13.70	2.48
1	7.06	0.23	0.98	2.56	0.08	20.90	23.70	14.90	4.21
2	7.07	0.25	1.73	3.16	0.09	26.50	28.40	16.60	5.65
5	7.12	0.27	0.94	3.57	0.22	62.00	48.40	21.80	10.60
8	7.10	0.33	3.61	3.96	0.38	99.70	64.80	27.20	15.60
10	7.11	0.36	2.06	4.07	0.49	151.00	85.50	36.70	20.00
12	7.14	0.39	3.87	5.08	0.54	174.00	96.30	39.70	22.70
15	7.01	0.44	5.20	7.04	0.57	225.00	121.00	47.50	29.00
18	6.95	0.49	6.42	7.65	0.78	255.00	140.00	53.70	33.90
20	6.91	0.52	7.16	8.86	0.76	293.00	153.00	58.00	37.50
100	7.57	1.58	7.51	41.90	5.88	423.00	335.00	160.70	128.40

Abbreviations: DOC, dissolved oxygen (mg L^−1^); EC, electrolyte conductivity (ds/m); TN, total nitrogen; TP, total phosphorus; VCT, vermicompost tea.

**TABLE 2:** Chemical properties of VCT at different concentrations on day 26 (*n* = 3), *p* < .01.

VCT (%, v/v)	pH	EC (ds m^−1^)	TN (mg L^−1^)	K+
Control	5.66D ± 0.04	0.08DF ± 0.00	2.33G ± 0.03	0.96F ± 0.17
1	5.53D ± 0.08	0.07DF ± 0.00	1.89G ± 0.43	1.38F ± 0.30
2	5.66D ± 0.14	0.15D ± 0.00	3.48G ± 1.07	0.60F ± 0.14
5	6.23C ± 0.37	0.25F ± 0.00	4.88G ± 1.07	2.21F ± 0.11
8	6.76B ± 0.04	0.82CD ± 0.00	7.58F ± 1.08	2.76F ± 0.07
10	7.02AB ± 0.08	0.70CD ± 0.17	18.96E ± 0.94	5.55E ± 0.22
12	7.10A ± 0.03	1.02C ± 0.08	45.07D ± 1.25	44.62F ± 0.30
15	7.02AB ± 0.11	1.90B ± 0.65	71.48C ± 0.30	81.77C ± 2.41
18	7.04AB ± 0.04	1.94B ± 0.21	133.00B ± 2.65	183.78B ± 3.07
20	7.09A ± 0.05	2.90A ± 0.58	142.00A ± 3.61	189.52A ± 0.70

### 3.2 VCT affect the growth of maple peas

**FIGURE 1:**
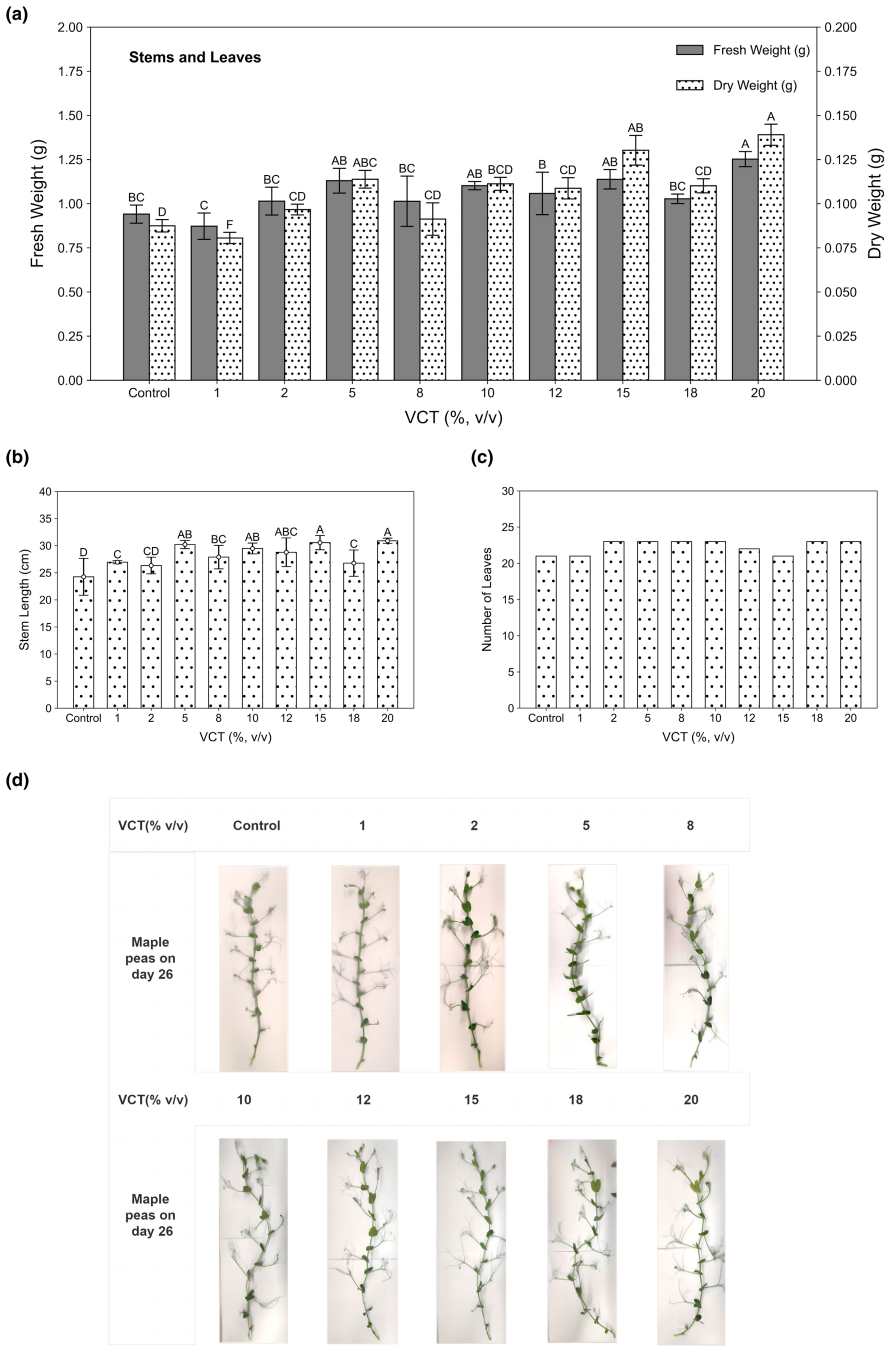
Growth of maple pea stems and leaves after 26 days (a) fresh and dry weights of stems and leaves, the gray bar is fresh weight, corresponding to the left coordinate scale (0.0–2.0), and the spotted bar is dry weight, corresponding to the right coordinate scale (0.0–0.2) (*n* = 30), (b) length of the main stem measured with ImageJ software (*n* = 10), (c) the number of leaves (*n* = 30), (d) representative growth images of maple pea stems and leaves at various VCT concentrations. VCT (%): 1, 2, 5, 8, 10, 12, 15, 18, 20 indicate the proportion of solutions added to VCT (of total volume), control: only water was added, *p* < .01.

The effect of VCT on maple peas is most visually evident in some of the plant growth indicators, such as the weight of the stems, leaves, and roots. 1% VCT showed an average weight of 0.87 g, only 0.07 g lighter than control (Figure 1a). 20% VCT had the heaviest weight of 1.25 g which was statistically significantly different (*p* < .01) from control. The dry weights with 5%, 15%, and 20% VCT were statistically significantly different (*p* < .01) from control.

In addition to fresh and dry weight, stem length and number of leaves also were compared to explore the effects on plant growth. Control group stem length was 24.25 cm (Figure 1b), whereas 20% VCT was 30.91 cm. Stems of 5%, 15%, and 20% of VCT treated groups grew to 30 cm, which is the growth limit of maple peas. All experimental groups except 2% VCT had statistically significant differences compared to control (*p* < .01). The average number of leaves of maple pea was 22, with no statistically significant difference (*p* > .01) from control. The morphology of the 10 maple pea plants shows that branch length increases with increasing VCT concentration (Figure 1d). This indicates that maple pea grows laterally after reaching its height limit, suggesting that high VCT concentrations have rich nutrients that promote peas growth.

**FIGURE 2:**
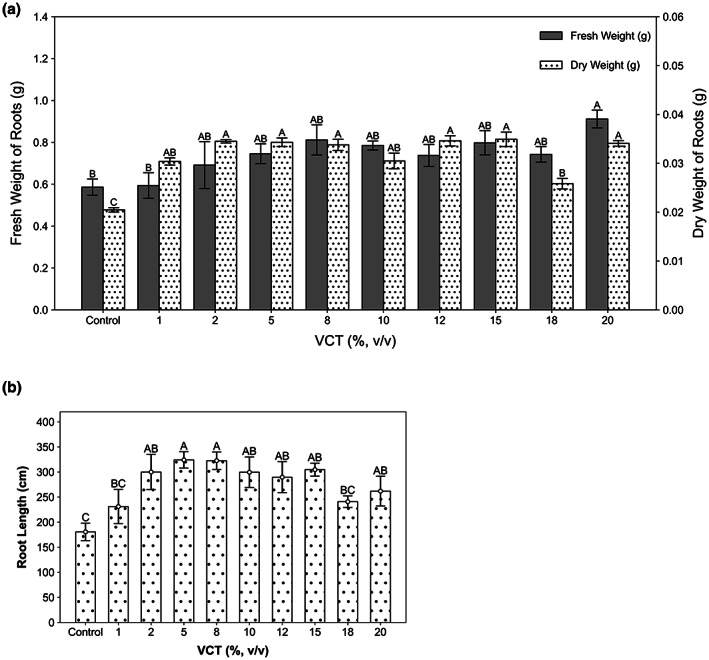
Figure 2 Root growth of maple pea after 26 days (a) gray bar is fresh weight, corresponding to the right coordinate scale (0.0–1.4), and spotted bar is dry weight, corresponding to the left coordinate scale (0.00–0.06) (*n* = 30), (b) total root length (*n* = 10). *p* < .01

The 20% VCT group had the heaviest fresh weight of 0.91 g (Figure 2a) and control had the lightest fresh weight of 0.59 g. However, only the 20% VCT treated roots had statistically significant difference (*p* < .01). Control group had the lightest dry weight of 0.02 g (Figure 2a) while the 20% VCT had the heaviest, and all experimental groups were statistically different (*p* < .01) from control. The weight difference between roots and stems and leaves is not strong. It shows that maple pea roots have well‐developed, possibly because VCT contains a lot of nitrogen. The longest root was 322.6 cm, and the shortest was 180.7 cm (Figure 2b). The 1% and 18% VCT had no statistically significant difference (*p* > 0.01) compared to control.

VCT enhances the nutrients and minerals needed for the plants, resulting in increased biomass and length of maple peas. Pant et al. (2011) reported that application of vermicompost tea increased plant yield, total carotenoids and total thioglucosides in plant tissues. Akinnuoye‐Adelabu et al. (2019) suggested the use of 5% and 10% VTC concentrations to improve the germination rate, optimal germination, and emergence of seeds. In this study, 20% VCT concentration was the most effective in promoting the weight of plant stems and leaves, while adding VCT was able to promote the growth of plant stems. It is demonstrated in the present study that VCT improved the mineral nutrient status of plants and enhanced the solution biological activity. Moreover, VCT can increase roots' weight, and medium concentration of VCT had the most favorable effect on root length. However, once the growth of roots was too vigorous, the nutrients transmitted to the stem and leaves were relatively reduced, which is detrimental to stems' and leaves' germination.

### 3.3 Effect of VCT on nitrogen content in maple peas

As mentioned previously, nitrogen plays many roles in plant growth, such as promoting chlorophyll synthesis, enhancing photosynthesis, providing adequate nutrients to plants, and promoting plant growth (Marschner, 2011). Nitrate and ammonium nitrogen are effective forms in the soil which can be directly absorbed and used by the root system, hence they are also known as fast‐acting nitrogen. Although they have similar nutritional effects on plants' growth, there are some differences in uptake and utilization (He & Liu, 1991).

**FIGURE 3:**
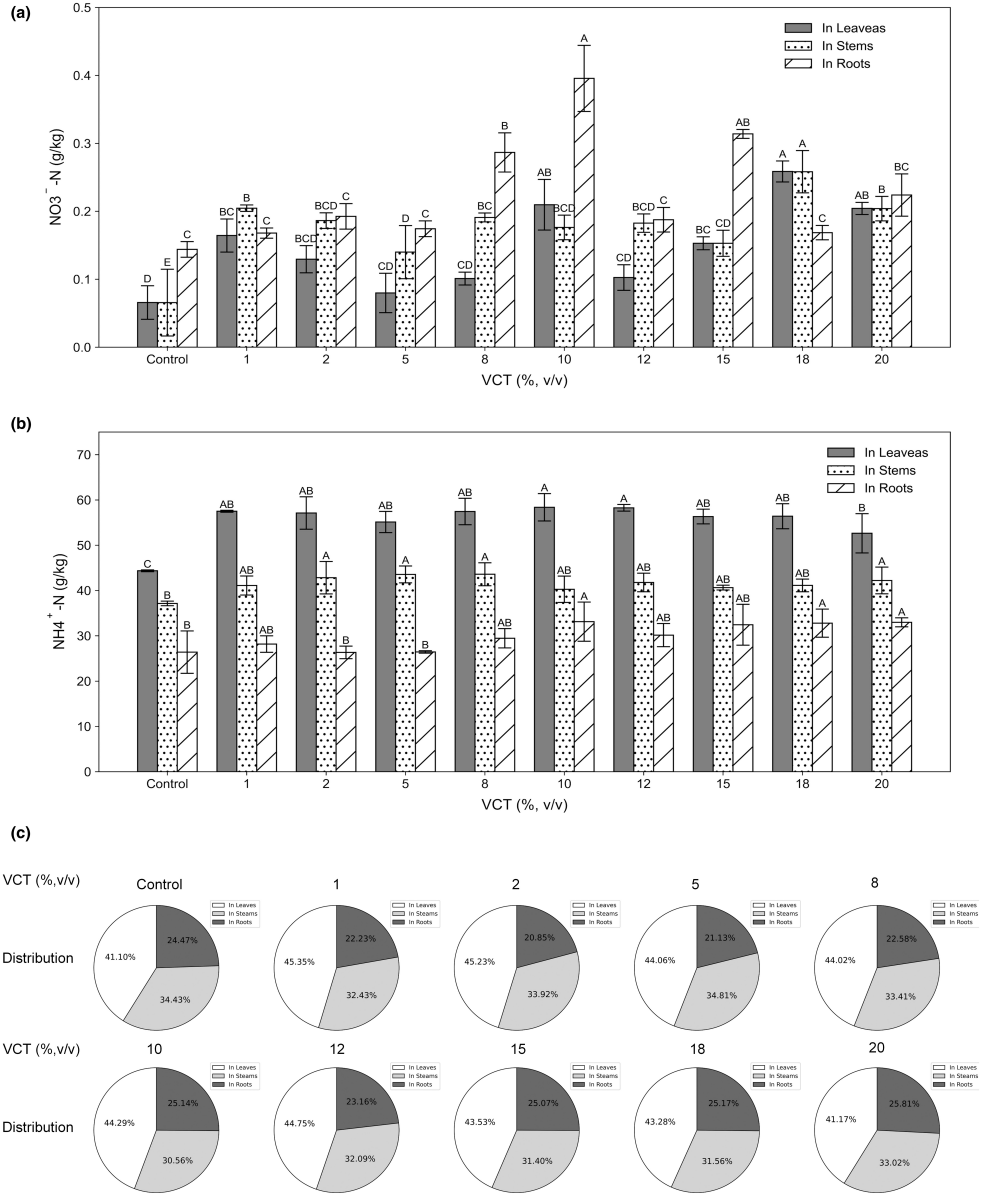
Figure 3 (a) NO3‐‐N in maple peas leaves (gray bar), stems (spotted bar), and roots (slash bar), *n* = 5 (b) NN4+‐ N in maple peas leaves (gray bar), stems (spotted bar), and roots (slash bar), *n* = 5, (c) the pie chart shows the distribution of NH4+‐N in the leaves, stems, and roots. *p* < .01.

In maple peas, N was mainly present as ammonium ions (NH4^+^) in the stems, leaves, and roots (Figure 3a,b). NO3^−^‐N was measured at 0.066 g/kg in control groups' leaves, and at 0.08 g/kg in the 5% VCT, which not significantly different (*p* > .01) from control. In 10% VCT, the NO3^−^‐N in the root was about 0.4 g/kg, and in control was only 0.14 g/kg. In comparison to control, roots treated with 8%, 10%, and 18% VCT had statistically significant increase (*p* < .01). Based on Figure 3a, it can be seen NO3^−^‐N is mainly found in roots, with little content found in leaves.

NH4^+^‐N content was above 40 g/kg in maple peas' leaves (Figure 3b), with the highest content in 10% VCT and the lowest in control at 58.38 g/kg and 44.37 g/kg, respectively, a difference of 14.01 g/kg. There was statistically significant difference (*p* < .01) between all experimental groups and control. As compared to control, the maximum NH4^+^‐N content was 43.60 g/kg in the 8% VCT stems, and 37.16 g/kg in control. Data from 2%, 5%, 8%, and 20% VCT had significant difference (*p* < .01). Specifically, 2% VCT roots had 26.34 g/kg, while 10% VCT had 33.14 g/kg. There was only statistically significant difference when comparing 10%, 18%, and 20% VCT with control. Pie charts were drawn to show the concentration of NH4^+^‐N in the roots, stems, and leaves (Figure 3c), and the concentration did not differ much between them. It is found mainly in leaves.

This result indicates that VCT is able to enhance the uptake of NO3^−^‐N and NH4^+^‐N in the roots, stems, and leaves, whereas NH4^+^‐N was the dominant form absorbed. It is noteworthy that 10% and 15% VCT are more effective than the others in promoting nitrate nitrogen in maple peas, as its content of nitrate nitrogen was significantly higher (*p* < .01) than that of control. When it comes to NH4^+^‐N absorption, the 20% VCT was found to have the greatest effect. Hence, the conclusion can be drawn that plants were able to absorb nitrogen better when the VCT concentration was high. This could also be attributed to the abundance of nitrogen in VCT, which made it possible for the plants to take up nitrogen to the fullest extent possible.

### 3.4 Other elements in maple peas

**FIGURE 4:**
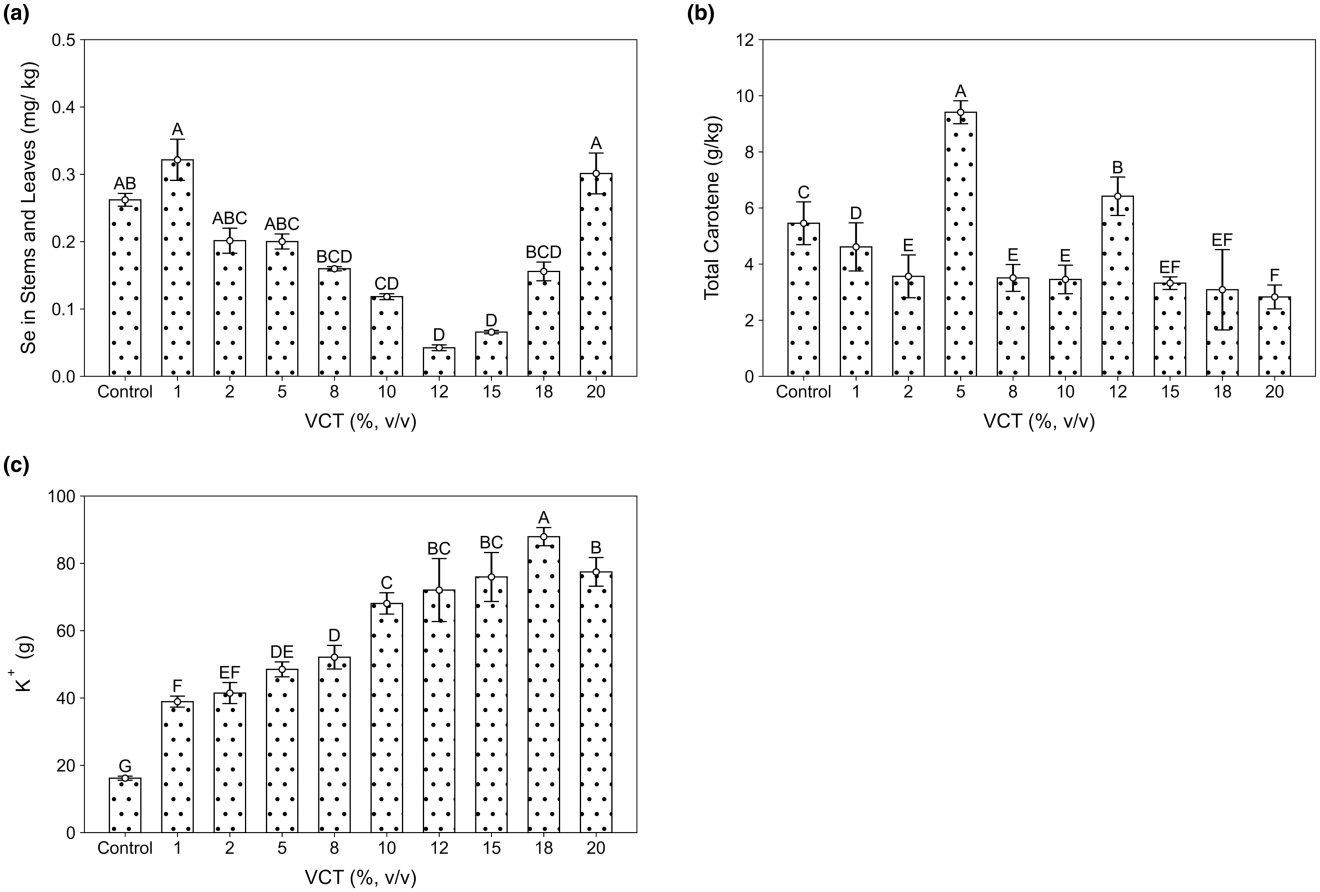
Detection of the nutrient content of (a) selenium in stems and leaves, (b) total carotene content in stems and leaves, and (c) potassium ion content (*n* = 5). *p* < .01

In addition to nitrogen, maple peas are also rich in carotenoids and Se, which can inhibit the production of nitrosamines in human, reduce the damage caused by cancer cells, and play a role in cancer prevention and anticancer. VCT at 12% (v/v) had the lowest Se content (Figure 4a), except for 1% and 20% VCT with higher Se content than control group, the rest of the experimental groups were lower, and not significantly different (*p* > 0.01). Total carotene content was highest in 5% VCT, and only 5% and 12% VCT were higher than control, which was significantly different (*p* < .01) (Figure 4b). Potassium is one of the main phytonutrients affecting plant growth and development; it is also involved in nutrient transport and uptake, and conferring resistance to abiotic and biotic stresses, thereby increasing yields of quality crops and providing resistance to plant diseases (Epstein & Bloom, 2005; Maqsood et al., 2013; Pettigrew, 2008). In this study, K^+^ content increased with increasing VCT concentration (Figure 4c), reaching its highest value at 18% VCT with 87.9 g, while its K^+^ content decreased at 20% VCT relative to 18%. There was a statistically significant difference (*p* < .01) between all experimental groups and the control. VCT is rich in K^+^ that can be fully absorbed by maple peas, affecting plant enzyme reactions, photosynthesis, and other functions, thus promoting the growth of maple peas.

### 3.5 Analysis of the bacteria community

Nutrients found in vermicompost include plant growth regulators and beneficial microorganisms such as bacteria, fungi, and actinomycetes (Jangra et al., 2019), while vermicompost has also been shown to significantly enhance soil biodiversity through improved microbial biomass (Hernandez et al., 2014). VC is rich in microorganisms, probably because the environment in the digestive tract of earthworm is conducive to the growth of microorganisms. The organic waste ingested by earthworm provides energy for microbial growth (Ravindran et al., 2016). Lu et al. (2022) experimentally confirmed that in the absence of UV blockage, VCT supplementation promoted nitrogen and mineral accumulation in mustard plant, increased beneficial bacteria, and reduced fungal pathogens. Therefore, adding VCT can influence the microbial community and promote plant growth.

However, the root system, which is the primary organ of plant nutrient and water uptake, is populated and surrounded by a complex community of microorganisms called the root microbiome (Hacquard et al., 2015). It is well established that interactions with the root microbiome have the potential to influence plant health and development (Berendsen et al., 2012; Panke‐Buisse et al., 2015), since microorganisms are key players in nutrient cycling and acquisition by plants (Bulgarelli et al., 2013; Mishra et al., 2012). The root microbiome is recruited from a variety of microorganisms present in the surrounding bulk soil (soil biota outside the root zone) (Lareen et al., 2016). In soils, several studies have shown that soil inter‐rhizosphere bacterial populations can beneficially affect many plants such as wheat, potato, maize, grass, pea, and cucumber by colonizing the inter‐rhizosphere (Cakmakci et al., 2006). Additionally, the root microbiome has been shown to correlate with the developmental stage of plants. Soil‐microbial‐root interiors form a fixed triangle that synergistically influences plant growth (Tilak et al., 2006). As aforementioned, through the detection of plant growth indicators and nutrients, we found that VCT can promote the biomass of maple pea and improve the absorption of N and K elements. Therefore, we wanted to investigate further the microbial composition of VCT and the changes in the inter‐root microbial community in order to elucidate whether the inter‐root microbial community can extend its reach into the water used in hydroponic cultivation and influence the maple peas' growth.

#### 3.5.1 Bacteria in VCT at different concentrations

**FIGURE 5:**
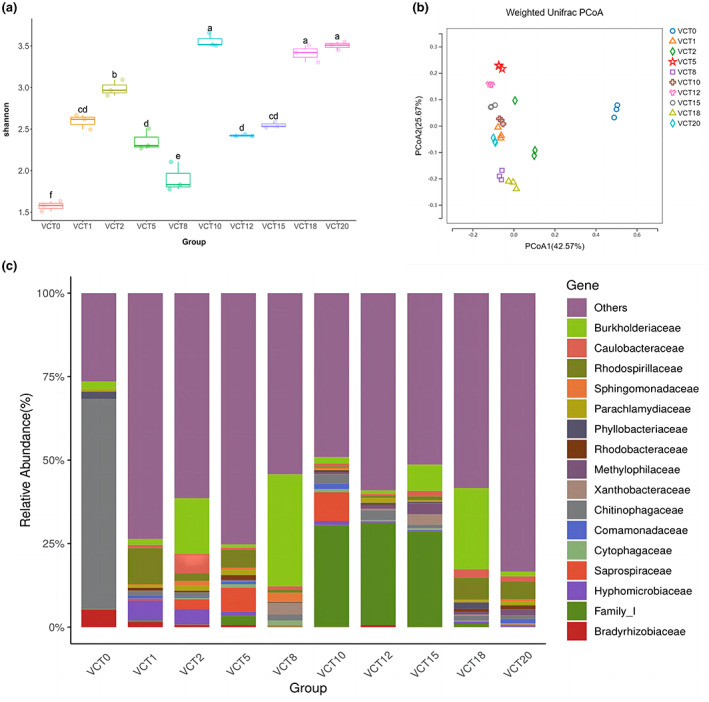
Bacterial abundance and phase dissimilarity of VCT at different concentrations on day 26 (a) Shannon index of microbial community. The horizontal bars within boxes represent medians, and the tops and bottoms of boxes represent the 75th and 25th percentiles, respectively. The upper and lower whiskers correspond to data no more than 1.5×the interquartile range from the upper edge and lower edge of the box, respectively. VCT0 represents control group, VCT1 represents 1% VCT, and so on. (b) Weighted Unifrac Principal Coordinate Analysis (PCoA analysis) plots, reflecting control and experimental groups’ phase dissimilarity between control and experimental groups. (c) Histogram of the species composition of the family‐level distribution of microbial community. Others—species with less than 0.5% species abundance and species not annotated to this taxonomic level (*n* = 3).

According to the Shannon index of alpha diversity, box line plots (Figure 5a) showed the diversity of bacterial communities in control (VCT0) was the lowest, while the diversity of the VCT20 community was the highest. With the Shannon index of diversity difference between VCT10, VCT18, and VCT20, there was no significant difference (*p* > 0.05). As VC is the organic matter decomposed by earthworm, their gut bacteria are expected to be present in VCT inherently, as well as decomposing microorganisms from the soil. As a result, all experimental groups showed significantly higher (*p* < .05) diversity than the control (Figure 5a). Principal coordinate analysis (PCoA) was used to elucidate the separation pattern of bacterial communities across groups such that their similarities and differences could be identified. Unifrac weighting accounts for taxa abundance and is therefore more sensitive to groups of rare abundance. Weighted Unifrac PCoA showed that the control and experimental groups were separated along the first principal coordinate (Figure 5b), indicating that the bacterial community composition of the experimental groups were significantly different (*p* < .05) from control. However, the distance between each experimental groups along the same coordinate showed only small variability, with the greatest differences in bacterial community composition between VCT5 and VCT18, as seen in the community composition histogram. In contrast to the composition of the bacterial community present in solutions, VCT enhanced the diversity of the bacterial community.

**FIGURE A1:**
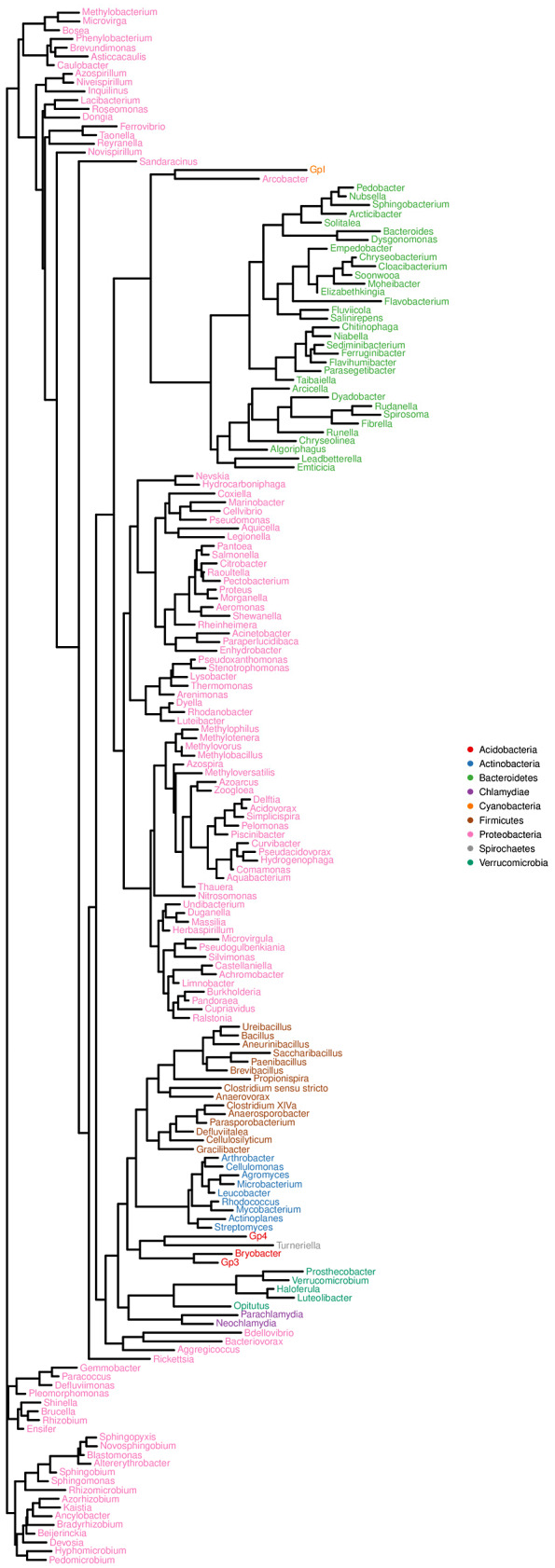
Species phylogenetic tree of VCT at different concentrations’ microbial community at the genus level, with the one with the highest abundance selected as a representative sequence for construction.

There were significant differences in the composition of the bacterial community in VCT at different concentrations. *Chitinophagaceae* as the dominant species in the control had the highest percentage (Figure 5c), reaching 62.7%, and was almost nonexistent in all experimental groups, and their percentage was all below 3% (*p* < .05). *Burkholderiaceae* was present in all groups with the highest percentage in VCT8 (reaching 33.5%). *Family_I* was accounted for about 30% in VCT10, VCT12, and VCT15, and less in control and the rest of experimental groups, with significant differences among them (*p* < .05). It is worth noting that *Family_I* belongs to *Cyanobacteria*, which are not currently identified in the Ribosomal Database Project (RDP), but it is clear that, based on the species phylogenetic tree, *Family_I* has the closest evolutionary relationship to *Arcobacter* (Figure A1).

**FIGURE A2:**
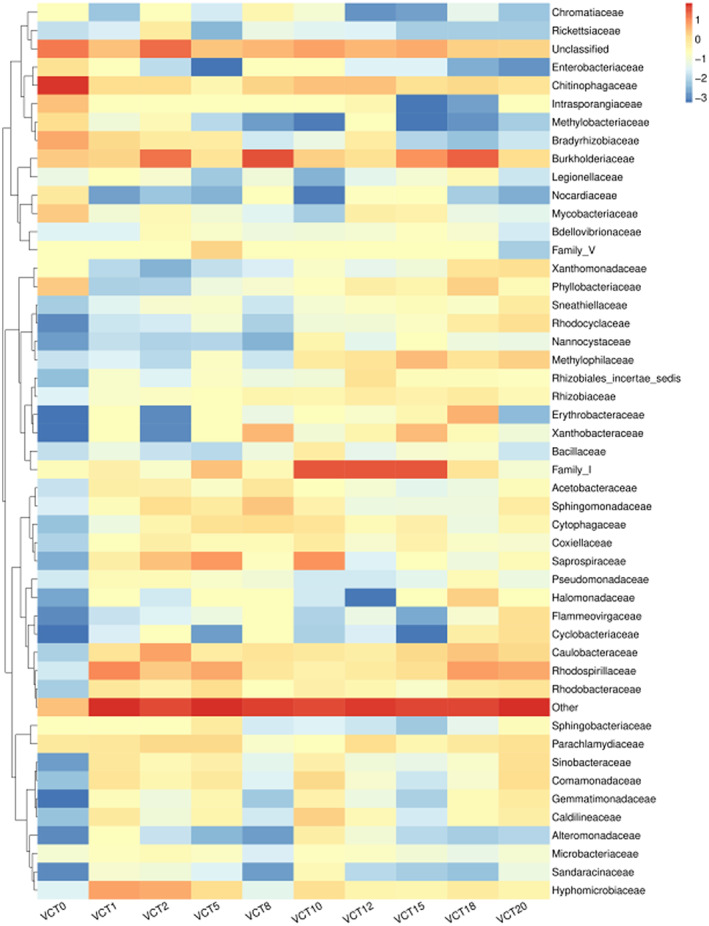
Species composition heat map, Others—species abundance all below 0.05.

*Rhodospirillaceae* is the dominant species in VCT1, VCT5, VCT18, and VCT20 (the highest percentage), and the species composition heat map makes it more obvious which families are more abundant in control or experimental groups, as well as the differences between them (Figure A2). *Rhodospirillaceae* can photosynthesize under the condition of light and hypoxia, and use light energy to assimilate carbon dioxide (Zhang & Sang, 1997). Not only does this provide a source of carbon for their own cell growth but also makes it available to other organisms through the food chain. In hydroponics, *Rhodospirillaceae* are in a hypoxic environment, so they would express more in the experimental groups so that they can obtain an additional carbon source. Similarly, *Rhodospirillaceae* have a fixation, assimilation, and degradation effect on organic objects as well as certain toxic substances in the water. Therefore, the dissolved oxygen content of the water is increased, improving environmental quality in hydroponics. According to Lu et al. (2008), it was found that *Rhodospirillaceae* can improve the yield and quality of hydroponically grown oilseed rape, probably because it can promote the uptake of iron by plants and increase the chlorophyll content of the crop, thus enhancing photosynthesis of the crop to improve crop yield (Liu & Xie, 2003).

#### 3.5.2 The bacterial community in the roots

**FIGURE 6:**
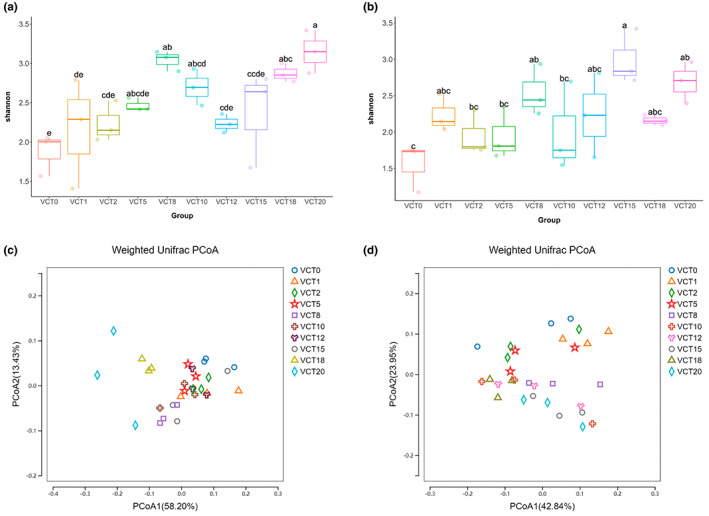
Bacterial alpha diversity and PCoA analysis of maple pea roots (a) Shannon index of the root microbial community on day 7, (b) Shannon index of the root microbial community on day 26, (c) PCoA analysis of root weighted units on day 7, (d) PCoA analysis of root weighted units on day 26. (*n* = 3)

Roots were taken for microbial community analysis at days 7 and 26, where day 7 was the stage when maple peas were grown to the point of edibility, and day 26 was the time when maple peas reached their maximum growth. It can be seen that the control had the lowest Shannon index for the bacterial community on both days 7 and 26 (Figure 6a,b), while VCT20 showed the maximum Shannon index on day 7, which was different from VCT18 that peaked on day 26 (Figure 6b). Although it is hard to draw a conclusive statement based solely on Shannon indexes, the results clearly indicates that the microorganisms in VCT remained highly diversified throughout the experimental period, with experimental groups showing significantly higher expressions on days 7 and 26 than control (*p* < .05).

The Weighted Unifrac PCoA analysis on days 7 and 26 showed that the differences between control and experimental bacterial communities surrounding plant roots were not as pronounced as in the solutions and were largely intermingled, indicating that the differences in species between the groups were not significant (Figure 6c,d). On day 7, except for VCT20 that was distant from the others, the rest of the experimental groups were relatively close to each other (Figure 6c). The VCT20 bacterial community was the most different from other groups, while the distance between its three samples were also far apart. Such an observation could be attributed to the small sample size which might not be sufficient to fully represent the complex bacterial community in the root. The shortcomings can be overcome with large‐scale studies. On day 26, the distribution of each group was discrete. There was no overlap between root control and experimental groups, and the relative distance between root control and VCT1 and VCT2 was the closest (Figure 6d).

**FIGURE 7:**
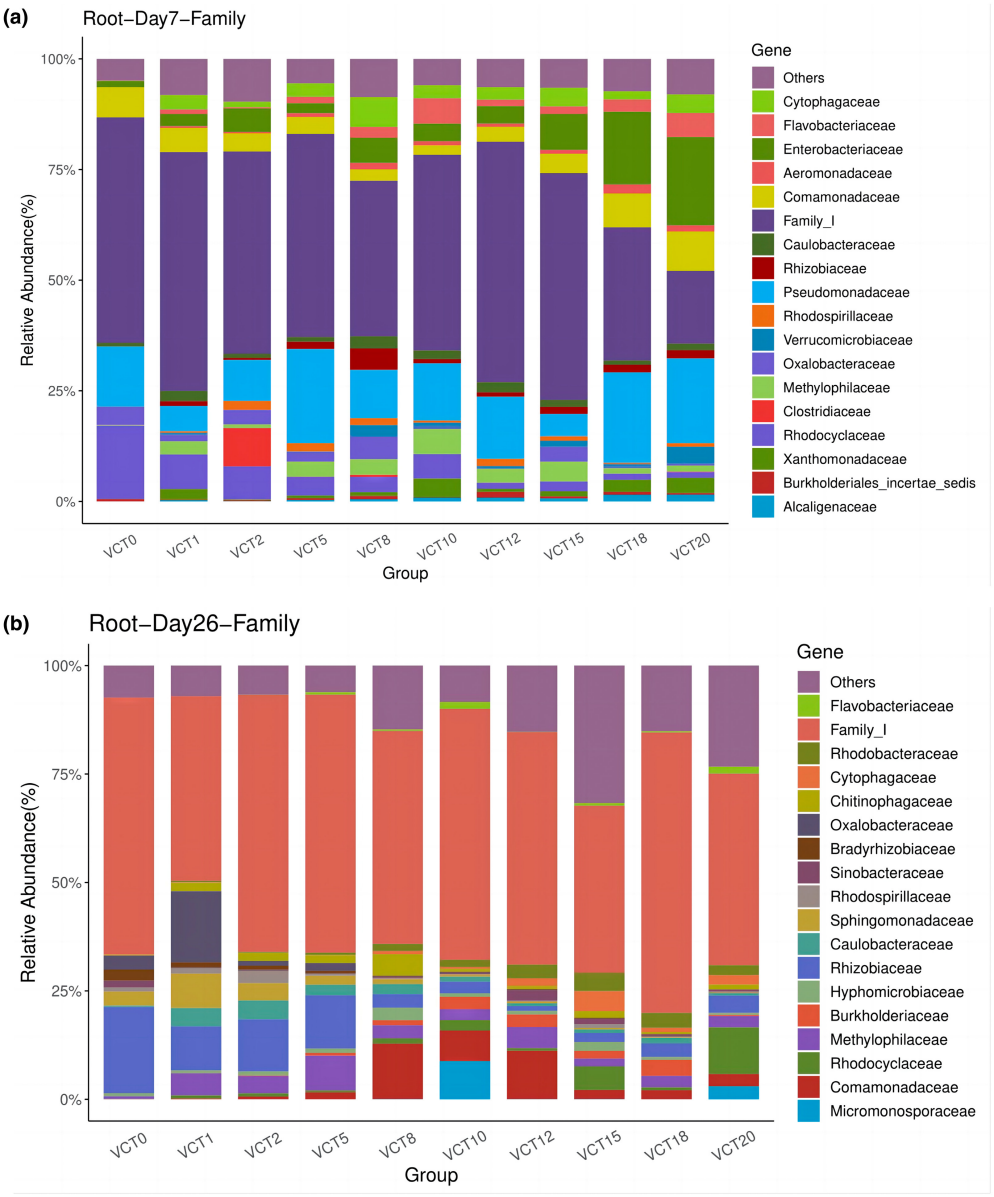
Species classification of OTUs by comparison with the database to obtain the family‐level distribution of microbial communities (a) on day 7 root systems and (b) on day 26 root systems, Others—species with less than 0.5% species abundance and species not annotated to this taxonomic level (*n* = 3)

**FIGURE A3:**
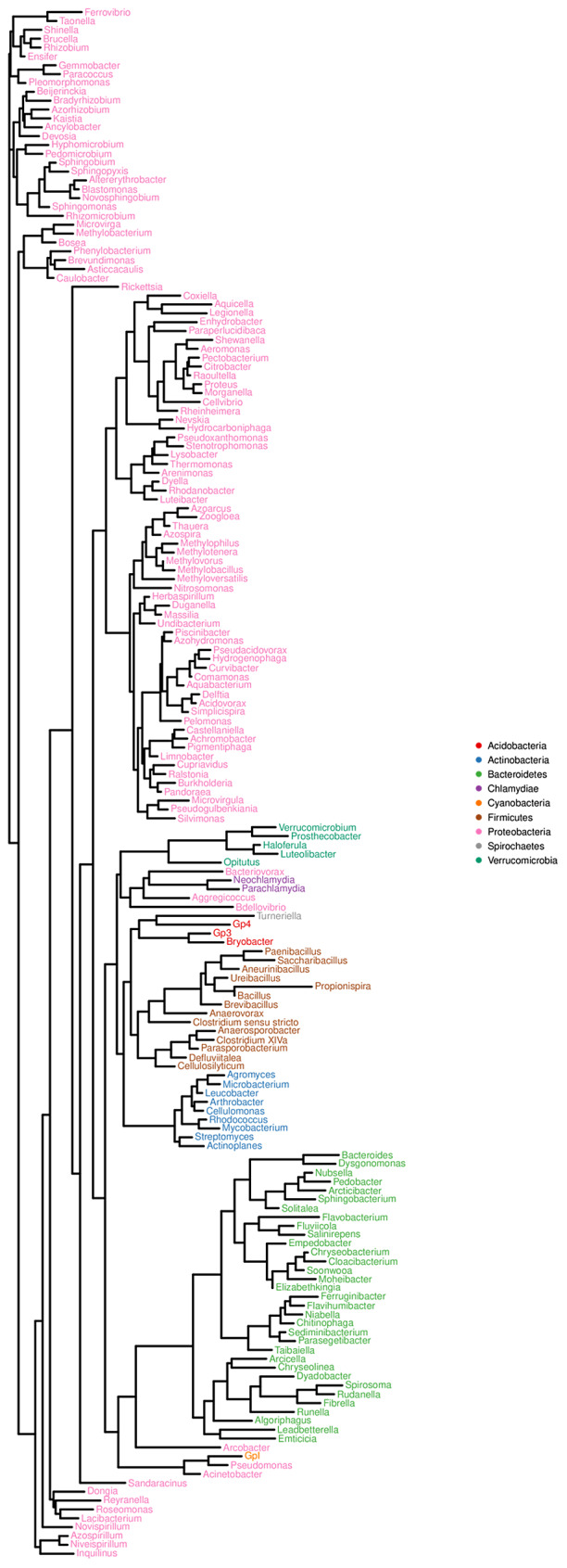
species phylogenetic tree of the root microbial community on day 7.

**FIGURE A4:**
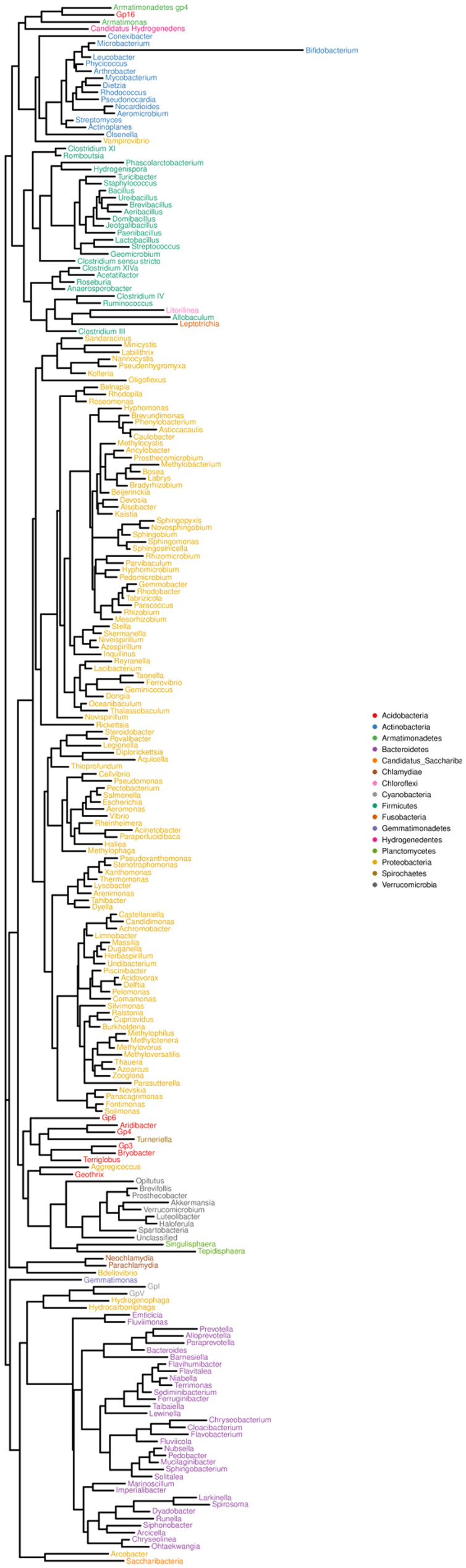
Species phylogenetic tree of the root microbial community on day 26.

The main bacteria in the roots on days 7 and 26 are *Family_I* (Figure 7a,b), which encompasses different types of microorganisms, but all belong to *Cyanobacteria*. *Family_I* on day 7 is evolutionarily related to *Pseudomonas* on the phylogenetic tree (Figure A3), while on day 26 was most closely related to *GpV* (*Leptolyngbya_foveolarum*) (Figure A4). *Pseudomonadaceae* were accounted for more than 5% of the root microbes in both control and all experimental groups on day 7 (Figure 7a), with VCT5 having the highest percentage of 21.3% and only 5.4% in VCT15. There was no significant difference between each group (*p* > 0.05). *Enterobacteriaceae* also occupied a large proportion in VCT18 and VCT20, reaching 16.4% and 19.9%, respectively. *Family_I* had the lowest rate of 16.5% in VCT20 and the highest rate of 54.4% in VCT12. According to statistical analysis, the difference of *Family_I* between VCT20 and control was significant (51.0%, *p* < .05). The highest percentage in control group, besides *Family_I*, was *Rhodocyclaceae*, which reached 16.6%. In the bacterial community of the roots on day 26, *Family_I* represented more than 35% in each group, with the highest expression of 64.7% in VCT18 (Figure 7b). The second highest expression of bacteria in control group was *Rhizobiaceae* which was also the most prevalent of all groups at 19.8%. *Rhizobiaceae* had a greater percentage in low (1%, 2%, 5%) than medium (8%, 10%, 12%), and high (15%, 18%, 20%) VCT concentrations. *Oxalobacteraceae* had the highest content in VCT1 with 16.4%, whereas the second most dominant bacteria in VCT20 was *Rhodocyclaceae* (10.7%).

**FIGURE 8:**
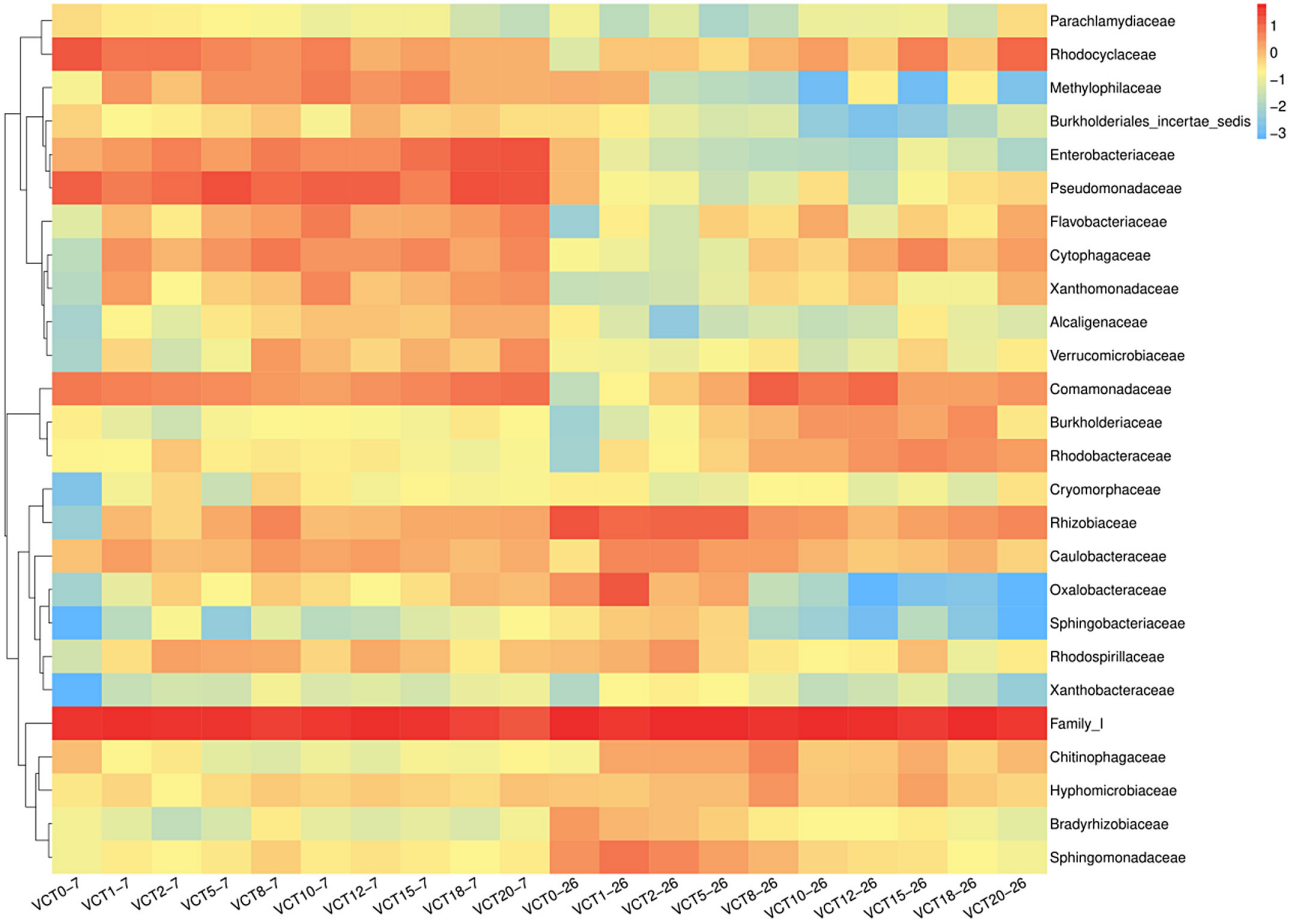
Comparison of days 7 and 26 species composition heatmaps, with the day 7 heatmaps on the left and the day 26 on the right. Blue indicates a relative abundance of ‐3 for the species and red indicates a relative abundance of 1 for the species. (*n* = 3)

The same bacteria on days 7 and 26 were selected to compare on a heat map, and it was evident from the map that bacterial communities drastically changed. The relative abundance of *Methylophilaceae* became lower in the experimental groups, while the opposite trend was observed in control (Figure 8), with VCT15 decreased the most from day 7 to day 26 (3.58). In freshwater ecosystems, *Methylophilaceae* are widely distributed and show a chemotactic response to ammonium (Dennis et al., 2013). The chemical analysis of VCT showed a significant level of total nitrogen (Tables 1, and 2), which may explain why the species declined substantially at a later stage.

The relative abundance of *Enterobacteriaceae*, *Pseudomonadaceae*, and *Flavobacteriaceae* was found to decrease with time, with *Enterobacteriaceae* decreased the most, from 1.3 to −2.0 in VCT20. *Enterobacteriaceae* are parthenogenic aerobic bacteria with respiratory and fermentative metabolism (Brenner, 1992), as well as being a common and major fermentative family in the earthworm gut (Wüst et al., 2011), so its relative abundance increased with increasing VCT concentrations in the early stages. However, the prolonged anoxic state of the aquatic environment in the later stages may be the main reason for the decrease in its relative abundance. *Pseudomonadaceae* and *Flavobacteriaceae* are the species that can be isolated from earthworm gut (Ihssen et al., 2003), and *Pseudomonadaceae* have functions such as stimulation of root growth, root repair, control of abiotic stresses, and plant diseases (Mendes et al., 2013). *Flavobacteriaceae* are common soil bacteria associated with cellulose degradation and contain several enzyme domains associated with fungal cell wall degradation (Carrión et al., 2019). They may interact with the roots in the early stages, serving to promote root growth and protect the plant from disease.

*Burkholderiaceae* decreased slightly in relative abundance over time in VCT1 (from −1.11 to −1.28), while it increased in the remaining experimental groups. *Burkholderiaceae* form nodules in legume roots, converting atmospheric N_2_ to plant‐available NH_3_ in exchange for the carbon compounds released by plants (Gyaneshwar et al., 2011; Oldroyd et al., 2011). *Burkholderiaceae* can promote plant growth activity (Chapelle et al., 2016), increase the abundance of microorganisms around plant roots, has beneficial interactions with plants (Aguirre‐von‐Wobeser et al., 2018), and can be positively correlated with biomass growth in soybean and alfalfa (Berg, 2009). The family may be a potential plant growth‐promoting rhizobacteria (PGPR) and accumulate at the inter‐root level by providing nutrients or signaling substances.

The proportion of *Rhizobiaceae* in the early stage was not high, and almost unseen in control. However, at the later stage, except for the decrease observed in VCT8, the relative abundance of *Rhizobiaceae* was increased in all other groups. The increase of *Rhizobiaceae* was most obvious in control (from −2.3 to 1.3) with three color gradients. *Rhizobiaceae* belong to the family *Rhizobia*, which Bergey's handbook describes as the family of soil bacteria that can form nitrogen‐fixing symbioses with legumes (Kersters, 1984), maintain legume growth in nitrogen‐poor soils (Garrido‐Oter et al., 2018), and provide plant growth factors and protect them from stress (Erlacher et al., 2014). Common *Rhizobia* or plant growth promoting rhizobacteria (PGPR) are present in the soil, but based on the above heat map (Figure 8), we found that *Rhizobiaceae* can survive and reproduce in water, subsequently supporting the growth of maple peas. The large increase of *Rhizobiaceae* in control is perhaps due to the stress on plant growth, with very few nutrients in the solution. Thus, to maintain the growth of maple peas, the signal molecules sent from the root interval make *Rhizobiaceae* proliferate. In contrast, the experimental groups had rich nutrients from VCT, so it did not increase massively and remained at a flat rate. Although the content of *Rhizobiaceae* in VCT8 was reduced, the relative abundance was still 0.5.

The *Sphingobacteriaceae* variation was not quite the same as the other families, with relative abundance increasing in control and low VCT concentrations while decreasing in medium and high. The members of the *Sphingobacteriaceae* are widespread and can be found in a wide variety of habitats, including soil, freshwater, etc. (Asker et al., 2008; Siddiqi et al., 2016), as well as animals' gut microbiota (Johnson et al., 2020). Some genera are well‐known as plant growth promoters that enhance crop yield, such as *Sphingobacterium* spp. and *Mucilaginibacter* spp., both can induce antioxidant systems and energy metabolism in plants, enabling them to cope with salinity‐induced toxicity (Vaishnav et al., 2020).

In summary, among the microorganisms present in the earthworm gut, *Enterobacteriaceae*, *Pseudomonadaceae*, and *Flavobacteriaceae* were detected in the inter‐rhizosphere of maple peas, indicating that the microorganisms in vermicompost can be retained in VCT by earthworm intestinal tract movement, excreting, and other vital activities. These microorganisms have the function of promoting root/plant growth and are present in large numbers. It is also possible to find microorganisms belonging to the *Rhizobia* family, *Burkholderiaceae* and *Rhizobiaceae,* which are known to be critical for legumes as they can form root or stem nodule symbioses. They also enter the host plant cells and differentiate into nitrogen‐fixing bacteria that interact host plant. *Rhizobia* are known to support symbiosis formation to produce growth hormone, vitamins, nitrogen fixation, and protection against plant stress. These findings are consistent with our chemical analysis that VCT‐treated maple peas had increased nitrate and ammonium nitrogen content relative to the control in roots, stems, and leaves.

## 4 CONCLUSION

VCT carries the desirable properties found in VC, such as rich nutrients and beneficial microorganisms. When added as a hydroponic solution to cultivate maple peas, VCT promoted its biomass and stem length, while increasing the content of K^+^ ions and N (mainly in the form of NH_4_^+^). While 15% VCT promoted the highest absorption of NO_3_^−^, 20% VCT promoted the absorption of NH_4_^+^ the best. VCT contains *Enterobacteriaceae*, *Pseudomonadaceae*, and *Flavobacteriaceae* that represent typical microbial activities of earthworm gut. The growth of maple peas was influenced by the presence of these species. Maple peas roots contained a number of strains that belong to the *Rhizobia* family as well, which can cause nitrogen fixation in the roots, which enhances the ability of nitrogen absorption and increases maple peas' biomass. Throughout the experimental period, both the abundance and species of the inter‐root bacterial population have altered, and these changes have affected the growth and nutrient uptake of maple peas. However, the mechanisms involved in these microbial balance changes remain to be explored.

## CONFLICT OF INTEREST STATEMENT

No conflict of interest.

## Data Availability

The data that support the findings of this study are available from the corresponding author upon reasonable request.
